# Toward Nitrite-Free Curing: Evaluation of a New Approach to Distinguish Real Uncured Meat from Cured Meat Made with Nitrite

**DOI:** 10.3390/foods10020313

**Published:** 2021-02-03

**Authors:** Juan de Dios Hernández, Ana Castell, Natalia Arroyo-Manzanares, Isidro Guillén, Pascuali Vizcaíno, Ignacio López-García, Manuel Hernández-Córdoba, Pilar Viñas

**Affiliations:** 1 Productos del Sur S.A. (Prosur), Av. Francisco Salzillo, P/27-2, San Ginés, 30169 Murcia, Spain; juandedios@prosur.es (J.d.D.H.); isidro@prosur.es (I.G.); pvizcaino@prosur.es (P.V.); 2 Department of Analytical Chemistry, Faculty of Chemistry, Regional Campus of International Excellence “Campus Mare-Nostrum”, University of Murcia, 30100 Murcia, Spain; ana.castell@um.es (A.C.); natalia.arroyo@um.es (N.A.-M.); ilgarcia@um.es (I.L.-G.); hcordoba@um.es (M.H.-C.)

**Keywords:** nitrite, nitrous oxide, cured meat, headspace gas chromatography–mass spectrometry

## Abstract

After the International Agency for Research on Cancer (IARC) classified ingested nitrites and nitrates as “probably carcinogenic to humans” under conditions favoring endogenous nitrosation, several meat products labeled as “made without nitrite” were launched. In order to distinguish uncured products truly made without nitrite from cured products made with any nitrite source (vegetal or mineral), this article presents an approach to detect and quantify nitrite from different origins added to meat. The method consists on the determination of nitrous oxide as a target compound using headspace gas chromatography–mass spectrometry (HS-GC–MS). Nitrous oxide (N_2_O) is formed after two reduction steps: from nitrite to nitric oxide (NO) and then to N_2_O. The NO is bound to myoglobin (Mb) or metmyoglobin (Met-Mb), forming a complex, which is subsequently released using sulfuric acid, which also favors the reduction to N_2_O. The HS-GC–MS conditions were split ratio 1:10; injection temperature at 70 °C; incubation temperature at 30 °C and time 45 min; and injection volume 1 mL. As a result, a relationship was established between the concentration of nitrite in cooked ham samples and the area of the N_2_O peak generated, meaning that this method allows the quantification of added nitrite within a concentration range of 10 to 100 mg kg^−1^.

## 1. Introduction

Nitrites have been used in the production of cooked meat derivatives as curing agents because their role in the development of a pink-red color and specific flavor profile and because of their bacteriostatic and bactericidal activity against some spoilage bacteria and dangerous foodborne pathogens. Nitrites prevent the growth of and toxin production by *Clostridium botulinum* and delay oxidative rancidity by chelating oxygen or reactive oxygen species (ROS) that promote oxidation, and also by forming nitrite and nitrosyl compounds that have antioxidant properties [1]. The European Commission classifies nitrites (potassium nitrite, E249; sodium nitrite, E250) and nitrates (sodium nitrate, E251; potassium nitrate, E252) as permitted food additives under Commission Regulation (EU) No. 1129/2011. The nitrite amount permitted as an additive in cured meat is currently 150 mg kg^−1^ (expressed as NaNO_2_). However, nitrites and their metabolic compounds are linked to potential adverse health effects. The main risks reported are the formation of metmyoglobin in blood, preventing the transport of oxygen through the body and the formation of N-nitroso compounds. In 2015, the Agency for Research on Cancer (IARC) classified ingested nitrates and nitrites as “probably carcinogenic to humans” under conditions favoring the endogenous nitrosation [2]. Although the nitrite amount established in cured meat does not involve a risk, excessive and prolonged nitrite consumption can affect human health. Myoglobin (Mb) is the main pigment in meat, and its forms determine the color changes that occur in meat. The deoxymyoglobin form is present inside of meat and has a purple color, while the oxygenated form (MbO_2_, Mb-Fe^2+^-O_2_) predominates in the meat surface and has a red color. Both Mb and MbO_2_ in their reduced forms provide the red color of meat. However, the oxidation of MbO_2_ leads to the complex metmyoglobin (Met-Mb, Mb-Fe^3+^), which is an unattractive brown in color. One way to stabilize the red color consists of adding nitrite anion (NO_2_^−^), which is reduced to nitric oxide (NO). The formation of NO is desirable because it acts as a ligand for binding to myoglobin and metmyoglobin (Met-Mb). Upon heating, nitrosylhemochrome species are formed from the dark-red NO complexes and have the pink-red color characteristic of cooked cured meat [3].

Nitrite can be used in cured meat products as sodium or potassium salts. The added nitrite is distributed in the cured meat between protein (20–30%), myoglobin (5–15%), lipid (1–5%), –SH (1–15%), as nitrate (1–10%), as nitrite (5–20%), gas (1–5%), and other species [4].

The nitrite anion can proceed from permitted food additives according to Commission Regulation (EU) No. 1129/2011, which must be labeled. Other sources of nitrite can be fermented vegetable extracts that are rich in nitrate, such as concentrated celery juice. Vegetable juices have been analyzed, obtaining amounts of 171, 2114, 2273, and 3227 µg g^−1^ of nitrate for carrot, celery, beet, and spinach juice, respectively [5]. Commercial celery juice powder was analyzed, and a nitrate content of 27,462 µg g^−1^ or about 2.75% was obtained, showing a higher concentration when drying [6]. The addition of nitrates leads to a prior reduction to nitrite by reducing bacteria, which makes the color stabilization slower. For long-life meat products, the addition of both nitrate and nitrite is necessary to ensure a continuous source of nitrite in meat over a long period of time. Frequently, meat products produced with these vegetable extracts are labeled “produced without nitrite” and this can mislead the consumer. The EU regulation establishes the permitted amount of additives that can be added to meat products but does not differentiate according to their origin.

Another way of stabilizing the color is by addition of natural antioxidants, mainly polyphenols, which are capable of regenerating Met-Mb to MbO_2_ [7,8,9]. Plant extracts have phenolic compounds, flavonoids, and organic acids, which have antimicrobial and antioxidant properties. These compounds can break the cell membrane, releasing the cellular components and affecting microorganisms [10] because they act as donors in the free radical chain reaction of lipid oxidation, thus showing antioxidant properties [11].

The analytical methods proposed for the monitoring of nitrite addition in meat are mainly based on the detection of residual nitrite by molecular absorption spectrophotometry using the Griess reaction [12] or high-performance liquid chromatography (HPLC) [13]. The Griess method is able to measure lower concentrations of nitrite than HPLC techniques; however, it shows low selectivity and may suffer from interferences. Moreover, it has been reported that residual nitrite disappears over time [14]. There is no relation between added nitrite with residual nitrite in the final product because, once it is added, the nitrite is transformed in NO and NO_2_ (nitrogen dioxide gas). NO forms nitrosyl or nitroso compounds (in combination with myoglobin and/or SH amino acid group), while NO_2_ in combination with water forms nitrous acid (residual nitrite) and nitric acid (residual nitrate) [15]. The regulatory aspects for food additives along with the correct application and interpretation of the norms have been recently discussed [16].

In this work, a procedure for the detection of nitrite from different sources in cooked ham samples is described. The method is based on that proposed for NO determination in tuna fish, as developed by Niederer et al. 2019 [17]. This method determines nitrous oxide (N_2_O) from the reduction of nitric oxide (NO), released from the complex NO–Mb. The novelty of the work presented here lies in it being the first application of the combination HS-GC–MS as an approach for the analysis of cured pork ham based on the determination of nitrous oxide from the nitrosylmyoglobin complex. The procedure permits, for the first time, establishing a methodology to distinguish real uncured meat from cured meat made with several sources of nitrite.

## 2. Materials and Methods

### 2.1. Reagents

Sulfuric acid (95–97%), 1-octanol, sodium chloride, and sodium nitrite were all from Merck (Buchs, Switzerland). Nitrous oxide gas (100% of purity) was supplied by Linde (Dublin, Ireland).

All headspace extractions were carried out in hermetically sealed 20 mL vials with PTFE/silicone septum caps of 20 mm (i.d.). Sodium nitrite was used to generate the nitrous oxide for the calibration standards using hermetically sealed vials for HS-GC–MS.

Polyphenol-rich extract (PRE) was obtained from PROSUR (Murcia, Spain), commercialized as NATPRE T-10 HT S. The NATPRE supplier reported less than 100 mg kg^−1^ in the product specifications. External analyses performed using normalized method EN 12014-7 shows values between 6 (±2) and 42 (±3) mg kg^−1^. This means that with a maximum recommended dosage (10 g kg^−1^), the possible ingoing nitrite in the meat is less than 1 mg kg^−1^ with no functionality in the final product.

### 2.2. Instrumentation and Software

A 7890A GC System gas chromatograph from Agilent Technologies (Palo Alto, California, USA), equipped with a programmable temperature vaporizer (PTV) model CIS4-C506 and an automatic injector (Headspace model Multipurpose Sampler MPS), both from Gerstel (Mülheim an der Ruhr, Germany), were used for HS-GC–MS analyses. Chromatographic separation was carried out on a HP-MOLSIV column with an internal diameter of 0.32 mm, a length of 30 m, and a film thickness of 12 μm, also from Agilent Technologies. The injection was performed in split mode with a ratio of 1:10. The GC temperature program was as follows: start temperature 70 °C, hold for 5 min, increase to 200 °C at 30 °C/min and maintain for 3 min; next, the temperature of 220 °C was reached at 3 °C/min and held for 5 min.

The detection was carried out using an Agilent 5973 mass spectrometer (Agilent Technologies) operating in the electron ionization mode (EI) at 70 eV, and temperature of the ion source was 230 °C. For the analyte identification, the selected SCAN mode was used, scanning from 10 to 100 *m*/*z* [15]. The selected ion monitoring (SIM) mode was used for quantification, the quadrupole was fixed at *m*/*z* 43.8; 29.8, and 28.0.

The HPLC with diode array detection (DAD) Agilent 1260, equipped with a Purospher^®^ Star RP-18 150 (5 µm) column, was used to determine residual sodium nitrite contents according to EN 12014–4 (2005).

Meat color determination was performed in a X-Rite 962 spectrophotometer (X-Rite Inc. Michigan, USA) using the D65/10° illumination/observation method.

Data were processed using Microsoft Office Excel (Microsoft, Washington, DC, USA), R Studio (version 1.2.5019) and MS data were acquired using Maestro 2 Version 1.4.25.8/3.5 software (GERSTEL).

### 2.3. Ham Processing and Sampling

#### 2.3.1. Preparation of the Cooked Ham Model System

Hams were produced at the PROSUR Meat Laboratory. Fresh pork ham muscles were received from a local processor and stored at 0 °C. The ham muscles were ground (CATO, Girona, Spain) to a size of 8 mm.

Cooked hams were prepared containing a 90% of meat, 1.5% of salt, 0.5% of phosphate, 8% of water, and different amounts of sodium nitrite (0, 0.5, 1, 5, 10, 20, 30, 40, 50, 60, 80, 100, 130 and 150 mg NaNO_2_ kg^−1^). In addition, ham samples containing NATPRE T-10 HT S with concentrations of 5, 10, and 20 g kg^−1^ and ham samples made with celery at 0.8 and 3 g kg^−1^ were also prepared. The final weight of each ham was 5 kg.

Non-meat ingredients were placed in a vacuum mixer (CATO, Girona, Spain) together with the ground ham and mixed for 1 h. The resulting meat–brine mixture (hereafter referred to as cooked ham model system) was transferred to a vacuum stuffer (CATO, Girona, Spain), packed in sausage casing and cooked until 68 °C core temperature (maximum oven temperature was 73–75 °C). Hams were chilled to 37 °C within 1.5 h and to 4 °C within 4.5 h. The weight after the chilling process was checked to assure that all brine was absorbed by the meat.

Afterwards, 500 g of the cooked ham model system was ground (Warning Commercial, Conair Corporation, Stamford, CT, USA), portioned in 10 samples of 50 g, and stored in polypropylene flasks with polyethylene caps at −25 °C until sample preparation (approximately one week).

#### 2.3.2. Commercial Meat Products

Commercial samples of meat products were obtained from local supermarkets. They correspond to four samples of cooked ham: cooked ham 3 and 4 were labeled as “nitrite free”, while no information about nitrite content was given for cooked ham 1 and 2. Two other samples correspond to a commercial *loin* dried and salt-cured and a serrano ham cured with salt, giving no information about the nitrite content.

### 2.4. Procedure for Residual Nitrite by HPLC–DAD

Residual sodium nitrite contents were determined according to EN 12014-4 (2005) by HPLC–DAD. Experiments were carried out for the nitrite-added ham samples and for the samples prepared with NATPRE T-10 HT S.

### 2.5. Color Measurement

For the color characterization (one week after preparation approximately), CIELAB color space was used. The parameters L* (lightness), a* (balance between green and red) and b* (balance between yellow and blue) were determined. A statistical analysis was performed after the color measurement of the different samples. Considering the a* parameter provided by the CIE L*a*b* technique, a discriminant analysis was carried out looking for significant differences between the different groups of matrices.

### 2.6. Analytical Procedure for HS-GC–MS

Meat samples were stored at −25 °C until sample preparation. For the analysis by HS-GC–MS, 15 g of sample was added into a 50 mL centrifuge tube with 5 g of crushed ice and 12 mL of water. The mixture was homogenized at 7000–9000 rpm with a polytron homogenizer (IKA, t25 digital Ultraturrax) and then centrifuged at 2500 rpm for 5 min at 10 °C. An aliquot of 8 mL of the resulting supernatant was transferred into a 20 mL vial and 8 μL of 1-octanol and 4 mL of sulfuric acid (20% *v*/*v* of concentrated acid solution) were added. The vial was immediately sealed and gently shaken by hand for 30 s.

The samples were incubated for the headspace analyses at 30 °C for 45 min, then injected into the GC for final detection by MS.

In order to minimize the effects of the matrix, the standard addition calibration method was used. Applying this methodology, the analytic signals for each of the samples is similarly affected by matrix interferences. Five cooked hams were prepared by adding increasing concentrations of sodium nitrite (10, 20, 40, 80, and 100 mg NaNO_2_ kg^−1^) for the calibration graph.

### 2.7. Validation Procedure

The following criteria were used for the validation of the procedure: calibration graph, linearity, accuracy, precision (repeatability and intermediate precision), limit of detection (LOD), and limit of quantification (LOQ). The quality control of the method was performed through routine analysis of procedural blanks as well as quality control of standards and samples.

The precision (repeatability and intermediate precision) was evaluated by analyzing the samples at three concentration levels (*k* = 3) in triplicate (*n* = 3) over 3 non-consecutive days (*p* = 3). The accuracy was assessed by control repetitions (3 per day) in three determinate levels (10, 30, 60 mg kg^−1^). The selectivity of the method was established by measuring the N_2_O concentration from real samples.

The LOD was assessed using the concentration value corresponding to three times the standard deviation of the estimate of the calibration graph. In the case of LOQ, the criterion was ten times the standard deviation of the estimate of the calibration graph. For confirmation, the LOD and LOQ values were assessed using a range of concentrations to the point of no detection or no quantification.

## 3. Results and Discussion

### 3.1. Evaluation of Color and Residual Nitrite Content

**Figure 1:**
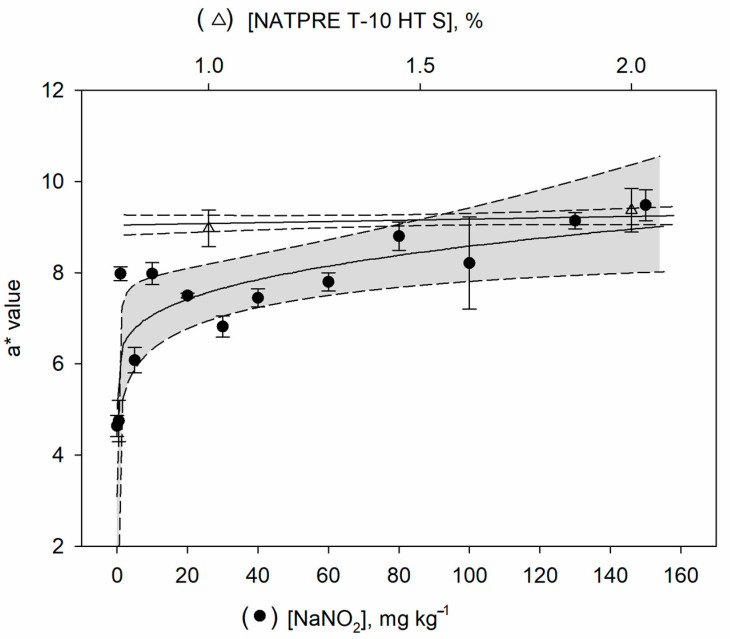
Influence of the presence of both sodium nitrite and NATPRE on the color of cooked ham samples.

The three forms of myoglobin (Mb, MbO_2_, and Met-Mb) may occur together in meat and have a characteristic visible absorption. NO–Mb has a similar spectrum to MbO_2_ [15]. To evaluate the cured meat color, the samples of cooked ham were divided into two groups, a group was treated with sodium nitrite and another group with NATPRE T-10 HT S. For each group of samples, three color replicate measurements were performed for each level of additive. These measurements were carried out at different sections of the sample to allow representative color values of each sample. The parameters L*, a*, and b* were determined, and the variable a* (red pigmentation) was used for comparison purposes. Figure 1 indicates that values of the variable a* increased for nitrite concentrations added to the cooked ham up to about 80 mg kg^−1^, while the signal remained practically constant for higher concentrations. However, the variable a* gave constant values for the uncured ham samples treated with NATPRE T-10 HT S. A *t*-test was applied to check if significant differences exist in the mean of the a* values between the groups when the maximum values were reached (from 80 mg kg^−1^ for the nitrite group). The results show that the means of the variable a* (red pigmentation) were not statistically significant at the 95% confidence level, with a *p*-value of 0.923.

These results indicate that the color of the product made with nitrite is similar to the color of the final product made with the NATPRE T-10 HT S, although in the first case, the color was produced by the direct bonding of nitrite, while for the ham made with addition of NATPRE T-10 HT S, the color of the product is mainly favored by the antioxidant activity of polyphenols, preventing the formation of Met-Mb [7,8,9]. Consequently, because the color measurements did not permit the differentiation of the cured and uncured ham samples, a new procedure is mandatory making possible to discriminate between both type of samples.

The determination of residual sodium nitrite content was carried out by HPLC–DAD on the same group of cooked ham samples. The results are shown in Table 1 and indicate that residual nitrite contents are higher when the concentration of added nitrite increases, whereas nitrite is not detected below LOD when 10 mg kg^−1^ or lower levels are added. On the other hand, no nitrite was detected below LOD in the ham samples treated with NATPRE T-10 HT S.

### 3.2. Optimization of HS-GC–MS Procedure

The parameters of split ratio injection, injection temperature, incubation temperature, incubation time, and injection volume were investigated in order to optimize the HS-GC–MS method using the SIM mode for the nitrous oxide ions. A cooked ham made with 100 mg kg^−1^ of sodium nitrite was used as sample to test the different instrumental parameters.

Preliminary experiments were carried out by volatilizing nitrous oxide from the aqueous solution to the HS. However, the obtained peaks were not defined. Consequently, a new approach including the addition of several microliters of a floating drop of an organic solvent was tried. 1-Octanol has been proposed as a preconcentration solvent for HS analysis of volatile compounds in aqueous matrices [18]. Thus, addition of 1-octanol at levels of 6, 8, and 10 µL was assayed, and maximum sensitivity was obtained when an 8 µL volume was added.

**Figure 2:**
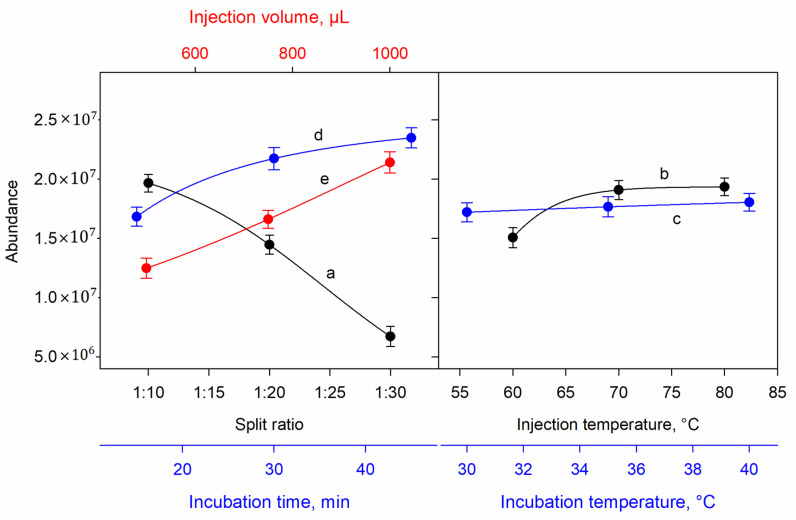
Effect of several experimental variables on the signal measured: (**a**) split ratio; (**b**) injection temperature; (**c**) incubation temperature; (**d**) incubation time; (**e**) injection volume.

The effect of split ratio between 1:10 and 1:30 was studied. Higher intensities were obtained with the lower dilution rate, as was predicted. Therefore, the split ratio was set at 1:10 (Figure 2a). The injection temperature was evaluated between 60 and 80 °C. As the temperature increased, the intensity of signals highly increased up to 70 °C, while this increment was very low for the temperature of 80 °C. Therefore, the optimal value was established at 70 °C (Figure 2b). The incubation temperature for the ham sample was optimized between 30 and 40 °C. An increase in both the baseline and the peak area were observed when the temperature increased, affecting the sensitivity of the method in the opposite way. Therefore, 30 °C was established as the optimal incubation temperature (Figure 2c). The sample incubation time was studied between 15 and 45 min. The longer incubation time was the optimal value. This can be explained by the longer time of extraction that facilitated the release of volatile organic compounds. Therefore, 45 min was chosen as the optimal incubation time (Figure 2d). Finally, the injection volume was optimized between 500 and 1000 µL. Higher peak areas were obtained when using the 1000 µL injection volume (Figure 2e).

### 3.3. Detection and Identification of N_2_O

**Figure 3:**
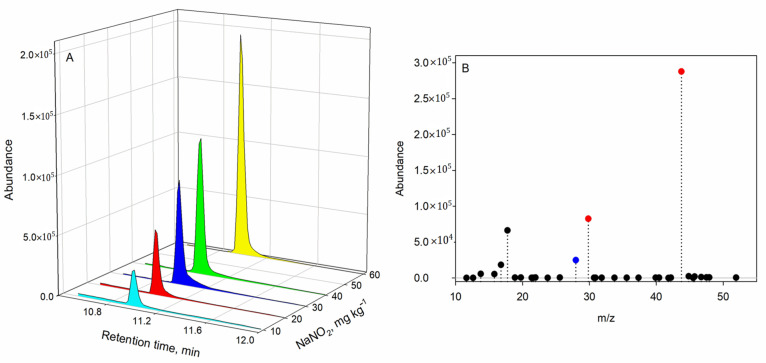
(**A**) Chromatographic profiles using headspace gas chromatography–mass spectrometry (HS-GC–MS) in selected ion monitoring (SIM) mode for different amounts of nitrite added to ham samples. (**B**) Mass fragmentation for nitrous oxide. Characteristic *m*/*z* values used were 43.8, 29.8, and 28.0.

In order to identify the nitrous oxide, two criteria were considered: the first was the addition of sodium nitrite when preparing the ham matrices; the results shows that the corresponding nitrous oxide signal increased when a higher sodium nitrite concentration was added, as shown in (Figure 3A).

The second criterion was to identify the analyte from the characteristic *m/z* ions. To identify the analyte, the SCAN mode was used for the quadrupole in a sample prepared with a concentration of 100 mg kg^−1^ of sodium nitrite. Then, the spectra were compared with the Wiley/NIRST mass spectral library (Figure 3B). The selected ions for quantification were fixed at *m*/*z* 43.8, 29.8, and 28.0. The ion at *m*/*z* 28.0 may also correspond to both nitrogen and CO [19], which could also be present, thus providing a blank signal value.

In order to ensure the identification of nitrous oxide by the proposed HS-GC–MS method, reference gas was measured in duplicate at six different injection volumes, from 0.1 to 3 µL, of pure nitrous oxide gas. A linear relation was obtained by plotting the peak area against the nitrous oxide concentration.

### 3.4. Validation of the Method

The HS-GC–MS method provided a linear relation between sodium nitrite concentration and the chromatographic peak area. Each point of the calibration graph was generated as the peak area of N_2_O within a concentration range of 10–100 mg kg^−1^ of NaNO_2_ added to the meat product. Calibration standards were prepared at five (*k* = 5) concentration levels of NaNO_2_: 10, 20, 40, 80, and 100 mg kg^−1^, each in triplicate (*n* = 3). The calibration graph was performed by plotting the peak area against the nitrite concentration, obtaining the following equation calibration: area = 5.4 × 10^5^ × nitrite concentration – 7 × 10^6^, *R*^2^ = 0.9932.

The accuracy was established by repetitive daily injections. As shown in (Table 2), trueness was found to be within an accepted value of −20% for the minor level and 4% for the major level. To detect random errors, the precision test was applied. Table 2 also shows the relative standard deviation values for repeatability (intra-day precision) and intermediate precision (inter-day precision) for each control sample concentration. As demonstrated, the relative standard deviation values for repeatability were between 3.6 and 5.2%, and intermediate precision values were between 7.3 and 10%.

The LOD value was assessed using the concentration value corresponding to three times the standard deviation of the estimate of the calibration graph (Sy/x = 176). Therefore, the LOD was 2.7 mg kg^−1^. The LOQ value was estimated as 9 mg kg^−1^. For confirmation, the LOD and LOQ values were assessed using a range of concentrations to the point of no detection or no quantification, values of 3.3 and 10 mg kg^−1^ being respectively obtained.

### 3.5. Method Application

The method validation was carried out by the analysis of NATPRE T-10 HT S (at 5, 10 and 20 g kg^−1^) and celery (at 0.8 g kg^−1^) cooked pork hams and nitrite-free hams. In order to study the variability of the samples, two different hams of each sample were tested. In addition, cooked hams with sodium nitrite added at different concentrations (10, 20, 40, 80 and 100 mg kg^−1^) were also measured. Table 3 shows the results of this recovery study. Sodium nitrite hams at different concentrations showed a good concordance between the amount of sodium nitrite added and that detected. These results ensure the accuracy of the proposed method.

To study the applicability of the method, different commercial samples of meat products obtained from local supermarkets were analyzed, corresponding to four samples of cooked ham and two samples corresponding to a loin and a serrano ham. The results obtained are shown in Table 4.

The products made with polyphenol-rich extract (NATPRE T-10 HT S), as well as nitrite-free hams, did not have a measurable level of nitrite, as expected. In the case of celery hams, the nitrate content is reduced to nitrite inside meat, which was measured by this methodology. In the case of the commercial samples, in cooked ham 1 and 2, the presence of nitrites was not explicitly shown in the ingredient list. However, nitrite appeared in both samples, one near the LQ and the other at a high nitrite level. The two samples cooked ham 3 and 4 were labeled as “nitrite free”, and the levels of nitrite found using the proposed method was very low, near the LQ level. The other two samples correspond to a loin and a serrano ham, giving no information about nitrite content, and for these samples, the levels of nitrite were not measurable by the method.

Therefore, the detection of nitrous oxide as a target compound using the proposed method provides an efficient way to identify nitrite treated meats from different sources [20]. A concentration of at least 100 ppm of nitrite is required for meat preservation, so LOQ at 9 ppm can be considered suitable for the identification of nitrite in meat as a preservative.

## 4. Conclusions

The method described presents a sensitive and selective approach for the quantification of nitrous oxide as a target compound, which is suitable for identifying nitrite-treated hams. A linear relationship was obtained between nitrite concentration in the sample injected in the headspace and the area of the nitrous oxide peak generated. Thus, a valuable approach was developed allowing, for the first time, distinguishing between uncured products which were truly made without nitrite addition and cured products made with addition of nitrite from different sources. NATPRE provides a very low concentration of incoming nitrite to the cooked hams, less than 1 mg kg^−1^. The antioxidant capacity provided by polyphenols is the key to being an alternative cure.

## Figures and Tables

**Table 1:** Residual sodium nitrite content determined by high-performance liquid chromatography with diode array detection (HPLC–DAD).

Sample	Sodium Nitrite (mg NaNO_2_/kg)
Nitrite (mg NaNO_2_/kg)	
5	ND ^1^
10	ND
20	3.7
30	5.8
40	5.6
60	8.5
NATPRE T-10 HT S (%)	
1	ND
2	ND

^1^ ND means No detected (below limit of detection at 2.7 mg kg^−1^).

**Table 2:** Trueness and precision (relative standard deviation, RSD %) (*k* = 3, *n* = 3, *p* = 3) studies.

Level (mg NaNO_2_/kg ham)	Relative Bias (%)	Repeatability (%)	Intermediate Precision (%)
10	−20	3.6	7.3
30	14	5.1	10
60	4	5.2	7.8

**Table 3:** Recovery study for sodium nitrite ham samples.

Concentration NaNO_2_ Added (mg kg^−1^)	Concentration NaNO_2_ Found (mg kg^−1^)
10	13 ± 5
20	17 ± 6
40	40 ± 4
80	89 ± 4
100	118 ± 5

Values are mean concentration ± standard deviation (*n* = 3).

**Table 4:** Sodium nitrite content in meat products determined by HS-GC–MS.

Meat Product	Concentration NaNO_2_ (mg kg^−1^)
NATPRE T-10 HT S (5 g kg^−1^)	NQ ^1^
NATPRE T-10 HT S (10 g kg^−1^)	NQ
NATPRE T-10 HT S (20 g kg^−1^)	NQ
Celery (0.8 g kg^−1^) ham 1	105 ± 3
Celery (0.8 g kg^−1^) ham 2	106 ± 4
Nitrite-free ham 1	NQ
Nitrite-free ham 2	NQ
Cooked ham 1	14 ± 1
Cooked ham 2	20 ± 5
Cooked ham 3	14 ± 6
Cooked ham 4	13 ± 1
Loin	NQ
Serrano ham	NQ

^1^ NQ means not quantifiable (below limit of quantification at 9 mg kg^−1^).

## Data Availability

Not applicable.
